# Phytochemical Constituents of Medicinal Plants for the Treatment of Chronic Inflammation

**DOI:** 10.3390/biom11050672

**Published:** 2021-04-30

**Authors:** Ki Sung Kang

**Affiliations:** College of Korean Medicine, Gachon University, Seongnam 13120, Korea; kkang@gachon.ac.kr

Chronic inflammation increases the risk of several serious human diseases. Inflammation is an acute physiological response caused by infection and tissue injury that results in the recruitment of plasma proteins and leukocytes to the afflicted tissue site, to eliminate the pathogen and initiate the tissue repair [1]. The tissue stress or malfunction induces an adaptive response of the immune system through tissue-resident macrophages called para-inflammation or chronic low-grade inflammation, which is the intermediate stage between basal homeostasis and inflammatory states [2]. If the stressful condition persists for a sustained period, para-inflammation could develop into a chronic state. The dysregulation of para-inflammation is responsible for the systemic chronic inflammatory states that are associated with numerous human diseases such as obesity, type 2 diabetes mellitus, cardiovascular disease, and neurodegeneration [2].

Plant-derived phytochemicals have emerged as novel agents for protecting against chronic disorders [3]. Owing to the diversity of phytochemicals, they cover a wide spectrum of therapeutic indications against cancer, inflammation, and neurodegenerative diseases, and have been a productive source of lead compounds for the development of novel medications. Actually, many of the effective drugs act via modulation of multiple targets rather than single protein [4]. In recent years, the pharmaceutical industry is facing challenges such as increased drug development costs, high failure rates, and increased competition for proven targets, and the demand for new target and pharmacological mechanism-based first-in-class drug development [5]. Recent attempts to introduce systems biological big data analysis and artificial intelligence (AI) technologies throughout the development of new drugs, such as drug virtual exploration, drug repositioning, new target discovery, bioavailability and side effect prediction, mode-of-action, and patient stratification for precision medical care, are being activated [6]. These systems biological and network pharmacological attempts can find some of their origins in the concept of traditional East Asian medicine (TEAM).

Unlike conventional medications, herbal mixtures in TEAM are based on a multicomponent and a multi-target approach derived from holistic philosophy [7]. In order to deal with complex diseases, multi component and multi target approach is potentially meaningful, despite the challenge to figure the complex mechanisms out. The “Kun-Shin-Choa-Sa” theory represents king–minister–assistant–ambassador for synergistic effects. In the past, ancient physicians thought that taking care of the human body is philosophically similar to running a nation. The “Kun” means a major medicine containing the main drug efficacy, and is supported by three different types of medicines, Shin (minister) boosting and complementing the Kun’s efficacy, Choa (assistant) reducing side-effects caused by the Kun, Sa (ambassador) facilitating the delivery of the Kun [8,9].

Like the “Kun-Shin-Choa-Sa” theory, earlier studies indicated that despite the intrinsic complexity of the herbal mixture, the medicinal plants have advantages of the synergistic interactions of their multi-components and their poly-pharmacological effects (Tarkang et al., 2016). Numerous phytochemicals contained in plants, each or together, exert the main drug efficacy, synergistically complementing the efficacy, and alleviating side effects. Since TEAM considers the human body as one, which is a complex interacting system, and each cell, tissue, organ is originally connected to each other, therapeutic drugs to restore the energy and the overall body balance are also prescribed by TEAM doctors according to the “Kun-Shin-Choa-Sa” theory [10]. Therefore, in order to understand the role of multi-components exhibiting complex efficacy, it is necessary to overcome the limitations of existing research methods.

Recently, efforts to understand the disease by integrating conventional molecular biological experiment, bio-big data analysis and AI technologies are increasing rapidly [11]. In order to know the poly-pharmacological effects of multi-components, a lot of time and effort is required only through conventional experiments. Therefore, it is possible to develop a deeper understanding of the disease and the development of better treatments through a more integrated approach. In particular, research of various phytochemicals, such as qualitative and quantitative analysis, and activity relationship is a cornerstone for drug development, and is an essential part of recent systems biology research. Consequently, establishment of new strategy through integrating conventional molecular biological experiment and recent AI technologies may make a great contribution to the identification of targets for disease treatment.

An important direction for future investigation is to further optimize the integrated phytochemical analysis and network pharmacology to predict multi targets. Combinatorial strategies which target multiple mechanisms, such as increasing anti-inflammatory efficacy and triggering a response of innate immune system, may offer the better chance for clinically meaningful treatment [10,12]. It will be necessary to adopt systems biology approaches along with omics techniques to explore the complexity of herbal medicines and phytochemicals on chronic inflammation and the underlying mechanism.

## Data Availability

Not applicable.
